# P-457. Impact of Switching to Bictegravir- or Dolutegravir-based Antiretroviral Therapy on Weight in People Living with HIV during the COVID-19 Pandemic

**DOI:** 10.1093/ofid/ofae631.657

**Published:** 2025-01-29

**Authors:** Nardine Karam, Monica Douglas, Stanley Moy

**Affiliations:** Long Island University, Staten Island, New York; Touro College of Pharmacy, Brooklyn, New York; SUNY Downstate Health Sciences University, Brooklyn, New York

## Abstract

**Background:**

Integrase strand transfer inhibitors (INSTIs) have been associated with weight gain with dolutegravir (DTG) and bictegravir (BIC) potentially carrying a higher risk than raltegravir (RAL) and elvitegravir (EVG). The COVID-19 pandemic has also been linked to weight gain. However, there are limited data that contextualize the impact of the COVID-19 pandemic on weight in people living with HIV (PLWH) receiving INSTIs. This study aims to evaluate weight change in PLWH who switched to DTG- or BIC-based antiretroviral therapy (ART) compared to those who remained on non-INSTI-based ART during the COVID-19 pandemic.

**Figure:**
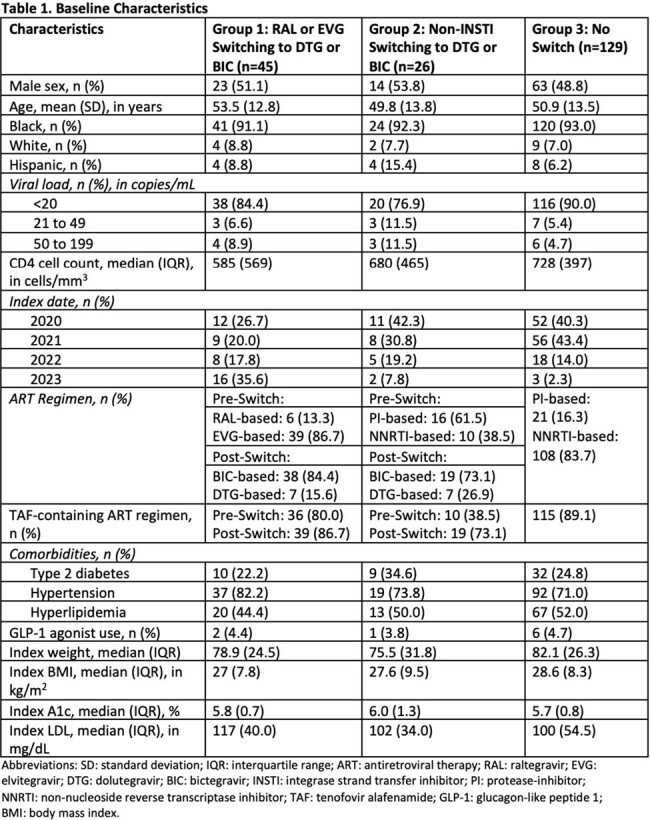

**Methods:**

This retrospective cohort study included virologically-suppressed PLWH seen at an HIV clinic between January 1st, 2020 to December 31st, 2023. Patients aged > 18 years were included if they switched from RAL- or EVG-based to DTG- or BIC-based regimens (Group 1), from non-INSTI-based to DTG- or BIC-based regimens (Group 2), or remained on non-INSTI-based regimens (Group 3). Pregnant and ART-naïve PLWH were excluded. The primary outcome was absolute weight change from index date to 6 and 12 months. The index date was defined as the switch date in Groups 1 and 2 and was based on the first weight available in the study period in Group 3. Secondary outcomes were absolute change in body mass index (BMI) at 6 and 12 months and in A1c and LDL at 12 months. Mixed linear models, adjusted for sex, age, and baseline BMI, were used to analyze the outcomes.

**Figure:**
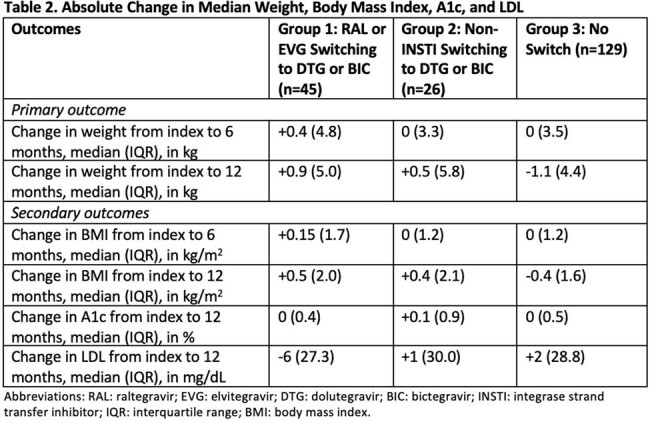

**Results:**

A total of 200 patients were included (n=45 in Group 1, n=26 in Group 2, and n=129 in Group 3). Baseline characteristics are presented in Table 1. Absolute changes in outcomes are summarized in Table 2. Groups 1 and 2 experienced a median weight increase of 0.9 kg and 0.5 kg, respectively, from index to 12 months, while Group 3 experienced a median weight decrease of 1.1 kg from index to 12 months. Model-generated means for outcome measurements are presented in Tables 3 and 4. Using mixed linear models, there were no significant changes in adjusted mean weight, LDL, and A1c between groups from index to 6 and 12 months (Table 3).

**Figure:**
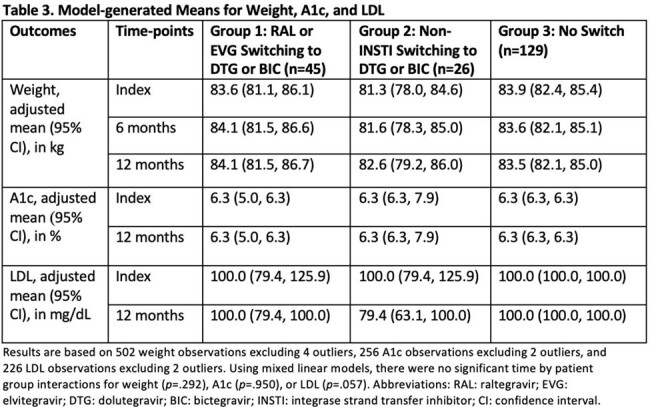

**Conclusion:**

Switching to BIC- or DTG-based ART during the COVID-19 pandemic was not associated with weight gain in treatment-experienced, virologically-suppressed PLWH in this single-center study.

**Figure:**
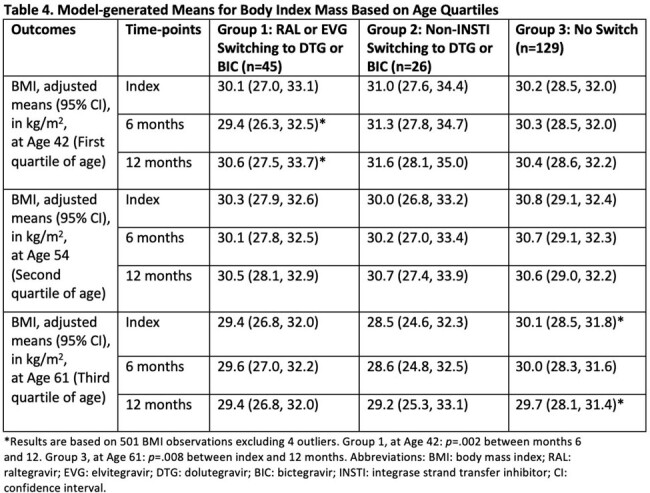

**Disclosures:**

**All Authors**: No reported disclosures

